# In Silico Design Strategies for the Production of Target Chemical Compounds Using Iterative Single-Level Linear Programming Problems

**DOI:** 10.3390/biom12050620

**Published:** 2022-04-21

**Authors:** Tomokazu Shirai, Akihiko Kondo

**Affiliations:** 1 Cell Factory Research Team, RIKEN Center for Sustainable Resource Science, 1-7-22 Suehiro-cho, Tsurumi-ku, Yokohama 230-0045, Japan; akihiko.kondo@riken.jp; 2 Department of Chemical Science and Engineering, Graduate School of Engineering, Kobe University, 1-1 Rokkodai, Nada, Kobe 657-8501, Japan

**Keywords:** FBA, metabolic model, optimization, AERITH

## Abstract

The optimization of metabolic reaction modifications for the production of target compounds is a complex computational problem whose execution time increases exponentially with the number of metabolic reactions. Therefore, practical technologies are needed to identify reaction deletion combinations to minimize computing times and promote the production of target compounds by modifying intracellular metabolism. In this paper, a practical metabolic design technology named AERITH is proposed for high-throughput target compound production. This method can optimize the production of compounds of interest while maximizing cell growth. With this approach, an appropriate combination of metabolic reaction deletions can be identified by solving a simple linear programming problem. Using a standard CPU, the computation time could be as low as 1 min per compound, and the system can even handle large metabolic models. AERITH was implemented in MATLAB and is freely available for non-profit use.

## 1. Introduction

Microorganism synthetic biology has been exploited to produce a variety of useful compounds [1,2,3]. In synthetic biology, genome-scale metabolic models (GEMs) are among the most powerful tools for the modification of intracellular metabolism with the aim of producing high amounts of useful chemicals. GEMs have been developed in several species [4], and they are available in public databases such as BiGG Models [5] and BioModels [6].

Flux balance analysis (FBA) is often used to modify cellular metabolism for the production of target compounds based on a GEM [7]. Concretely, FBA constrains metabolic networks based on the stoichiometry of metabolic reactions, and it does not require kinetic information. Using this approach, target compound yields are optimized by linking cell growth to the production of the target compound, thus maximizing both cell growth and target compound production. To achieve this, genetic modification of cells, including gene knockout and gene upregulation and downregulation, is necessary. The FBA is commonly combined with in silico screening to narrow down the candidate genes for genetic modification and decrease computation times, and various algorithms have been proposed for this purpose [8,9,10,11,12,13]. Among these approaches, OptKnock [14] is the most commonly used screening algorithm in both academia and industry, and it has been used to optimize the metabolism of various useful compounds produced by microorganisms [15,16,17,18]. This method is based on a bi-level linear programming approach and involves discrete decisions on reaction knockouts with binary variables, resulting in mixed integer linear programming problems (MILPs). This method can theoretically identify the most promising reaction knockout to achieve the highest target production yield among all possible sets of reaction knockouts. However, the number of reaction combinations increases exponentially as the total number of reaction knockouts increases, resulting in prohibitively long computation times. Therefore, the maximum number of reaction knockout candidates must be selected in advance to effectively implement this strategy. Realistically, several knockouts and approximately 100 candidates must be screened in advance for reaction defects. To achieve this, the FastPros algorithm, which introduces the concept of shadow pricing, excludes metabolic reactions that are not expected to affect the production of the target compound from the search using the *i*AF1260 model of *Escherichia coli* [19] paired with the OptKnock algorithm [20]. This enables the identification of effective combinations of more than 10 metabolic reaction deletions. Additionally, genetic design through branch and bound (GDBB), a heuristic approach that applies a branch-and-bound algorithm, is incorporated into the bi-level optimization framework used in OptKnock to identify near-optimal solutions in a matter of seconds or minutes instead of days or even longer [21]. Nevertheless, the bi-level optimization of the FBA model must be converted into a single-level MILP problem by introducing a dual problem to solve the complex MILPs. To perform these complex calculations and obtain accurate solutions, dedicated and commercial solvers are often required (CPLEX, ILOG Inc. [22], Gurobi Optimization [23]). In any given environment, cells are constantly adapting to maximize their growth; however, this does not necessarily maximize the production of target compounds. In fact, it is often the case that the production of the target compound and growth maximization cannot be confirmed using a combination of reaction defects obtained by executing OptKnock. Therefore, a combination of reaction defects that render the desired compound should be proposed using only growth maximization as the objective function.

In this study, we developed an algorithm to identify candidate reaction deletions that can potentially result in the production of large amounts of a target compound by simply iterating single-level linear programming problems. Furthermore, the proposed algorithm can be easily implemented using freely licensed solvers such as glpkmex. The iJO1366 GEM (i.e., a representative *Escherichia coli* GEM) was used to evaluate the effectiveness of the proposed algorithm for the production of various useful compounds.

## 2. Materials and Methods

### 2.1. Genome-Scale Metabolic Model (GEM)

The iJO1366 GEM [24] of *E. coli* was used as a metabolic model to validate and evaluate the algorithm developed in this study. Before executing the proposed algorithm, the reactions that were not deletion candidates, according to in silico screening, were removed. See Section 2.2 and Section 2.3 for more details on the computational calculation methods used here. Reactions associated with intracellular cytosol exchange and periplasmic space for transport were excluded as candidates for deletion. In contrast, the ABC system-based transport reaction, which was identified as a gene–protein reaction (GPR), and the phosphotransferase system (PTS) reaction were identified as candidates for deletion. The reactions that were not identified as deletion candidates are summarized in Supplementary Table S1. Of a total of 2583 reactions in the iJO1366 model, 1688 reactions, excluding 895 reactions related to transport, were considered deletion candidates for in silico screening.

### 2.2. Flux Balance Analysis

Metabolic design was conducted using constraint-based FBA in this study. Constructing a mathematical model for metabolic networks enabled the prediction of various functional metabolic states. Assuming that intracellular metabolism is in a pseudo-steady state, the rate of production and consumption of each intermediate metabolite was considered to be equal and therefore intermediate metabolites were not accumulated. Model constraints were then established, including specifying the range of possible solutions for each metabolic reaction flux.

(1) $$\overset{M}{\underset{j=1}{\sum }}S_{ij}v_{j}=0\;\;\;\;\;\;\;\;\;\;\;\forall i\;\in N\;v_{glc\_uptake}\leq \;GUR_{max}\;v_{o2\_uptake}\;\geq OUR_{min}\;v_{atp}\geq NGAM\;v_{growth}\geq \mu_{min}\;v_{upperbound}\geq v_{j}\geq 0\;\;\;\forall j\in M_{irrev}\;\;\;\;v_{upperbound}\geq v_{j}\geq v_{lowerbound\;\;\;\;\;\;\;\;\;\;\forall j\in M_{rev}\;\;\;\;\;\;\;\;}$$

where *S* represents a stoichiometric matrix in which *S_ij_* corresponds to the stoichiometric coefficient of metabolite *i* in reaction *j*; *v_j_* represents the flux of reaction *j*; and *M* and *N* are the reaction and metabolite sets, respectively; *v_glc_uptake_*, *v_o2_uptake_*, *v_atp_*, and *v_growth_* are the glucose uptake rate, oxygen uptake rate, ATP requirement for cell vitality, and growth rate, respectively; *R_irrev_* and *R*_rev_ are the irreversible and reversible reaction sets in the metabolic model, respectively; and *GUR_max_* and *OUR_min_* are the maximum glucose uptake and oxygen uptake rates, respectively. *GUR_max_* was set to 10 mmol/gDCW/h to compare the results calculated in this study with those in other studies. *OUR_min_* was set to the OUR value obtained by executing the FBA with the target compound as the objective function. *NGAM* represents non-growth-associated ATP maintenance, and was set at 3.15 mmol/gDCW/h as described in a previous study [24]. *μ_min_* is the minimum value of the specific growth rate, which was set to 0.05 h, as described by Ohno et al. [20]. Using these constraint equations, an objective function was established to conduct linear programming problem calculations and obtain the solution for each metabolic flux.

### 2.3. In Silico Screening

The following evaluation equation was introduced to rapidly screen for candidate deletions in metabolic reactions:

(2) $$chg_{j}=\frac{\left|v_{j}^{T_{max}}\right|-\left|v_{j}^{G_{max}}\right|}{\left|v_{j}^{G_{max}}\right|}$$

where *v_j_^Tmax^* is the flux value when the objective function is set to the target compound and FBA is executed; *v_j_^Gmax^* is the respective flux value when the objective function is set to the biomass growth rate and FBA is executed. To avoid alternative production fluxes acting as indeterminate solutions, the production flux of the target compound was maximized with the biomass growth rate, which was fixed at its maximum value. Specifically, the element corresponding to the production of the target compound in a column array containing the objective function coefficients was set to 10^−5^. *chg_j_* can be defined as the rate of change in the flux value of reaction *j* when the target compound is used as the objective function, as compared with that when the biomass growth rate is used as the objective function. *chg_j_* can take the following values, with −1 being the minimum value:

$$chg_{j}=-1\;:\;v_{j}^{T_{max}}=0\wedge v_{j}^{G_{max}}\neq 0\;-1<chg_{j}<0:\;v_{j}^{T_{max}}\neq 0\wedge v_{j}^{G_{max}}\neq 0,\;\left|v_{j}^{T_{max}}\right|<\left|v_{j}^{G_{max}}\right|\;chg_{j}=0:\;v_{j}^{T_{max}}=v_{j}^{G_{max}}\;chg_{j}>0:\;v_{j}^{T_{max}}>v_{j}^{G_{max}}$$

when *v_j_^Gmax^* is zero, *v_j_^Gmax^* is set to 10^−6^ to prevent zero division.

As *chg* approaches its minimum value of −1, the corresponding metabolic reaction is more likely to be a candidate for deletion. However, for reactions that are used for cell synthesis, the value of *chg* is always greater than −1 because *v_j_^Tmax^* can never be zero. To allow for the deletion of reactions that are preferentially used for cell synthesis but are not essential for cell growth, the following index was introduced:

$$ko_{r}=r\times chg^{min},\;0<r\leq 1$$

From the above equation,

$$chg^{min}\leq chg_{j}\leq ko_{r}$$

**Figure 1:**
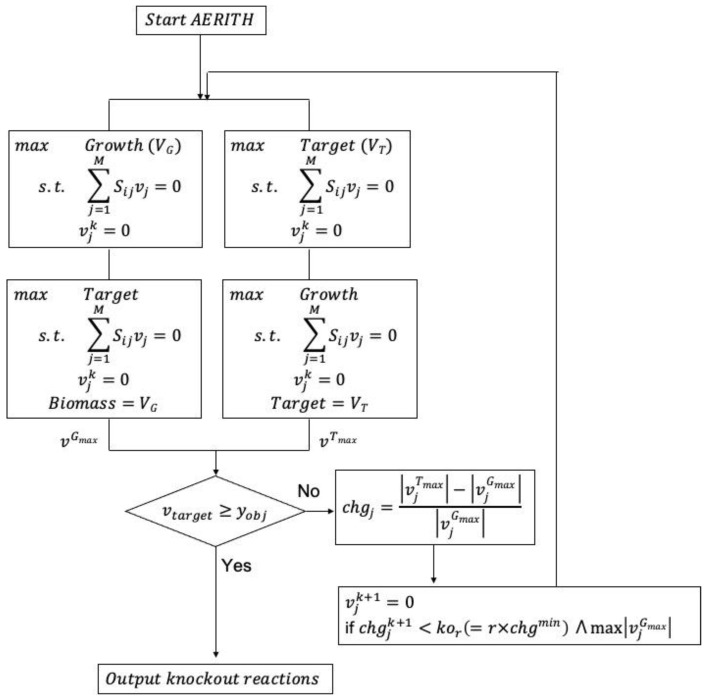
AERITH algorithm for target compound production. The objective function was set to maximize biomass growth and target compound, and two types of FBAs were performed. The value of *chg* was calculated for each calculated flux, after which the next deletion candidate was selected. *v_j_^k^* represents the kth deletion reaction and was added as a constraint to the FBA. *y_ob_*_j_ represents the minimum production flux of the allowed target compound.

A reaction *j* with the minimum value of *chg_j_* satisfying this condition was selected as a candidate for the deletion reaction. When the *chg* values were the same, the one with the highest *v_j_^Gmax^* was selected as the deletion candidate. In this study, all calculations were conducted using an *r* value of 0.95. The constraint condition expressed by $v_{j}=0$ was added to the constraint equation in Equation (1) and FBA was performed with the objective function set to the target compound and biomass growth rate. On the basis of the calculation results, a new *chg* was calculated using Equation (2), after which the next candidate for the deletion reaction was selected. A set of deletion reactions in which the production flux of the target compound increased and approached the theoretical maximum value (*v^Tmax^*) was thus obtained by repeating the simple linear program described above. The above-described iterative algorithm using single-level LP is shown in Figure 1. The proposed algorithm was named AERITH: algorithm of efficient reaction identification for target compounds with high productivity. The Cobra toolbox [25] was used to load the GEM before running AERITH, and all calculations, including linear programming problems, were performed using the GNU Linear Programming Kit [26] and MATLAB 2020a (MathWorks, Inc., Natick, MA, USA).

## 3. Results and Discussion

**Figure 2:**
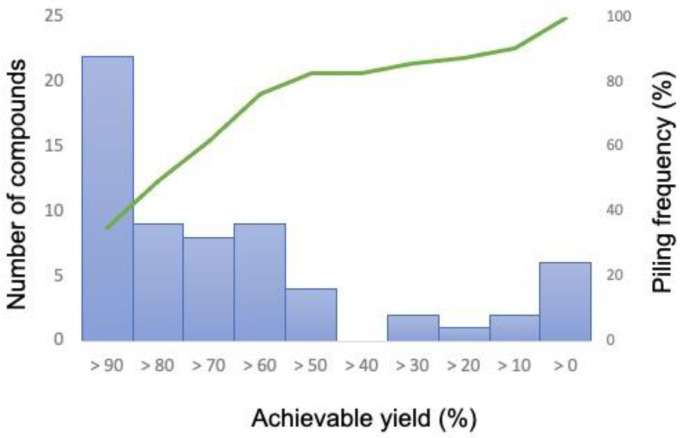
Distribution of the number of compounds that achieved production yield (left axis) and piling frequency (right axis) upon executing the AERITH algorithm. The percentage of yields that can be achieved with the combination of the deletion reactions is represented in units of 10% with respect to the theoretical production yields of each compound (Supplementary Table S2). The frequencies of the number of compounds satisfying these yields and the frequencies by accumulation from high productivity are also shown.

**Figure 3:**
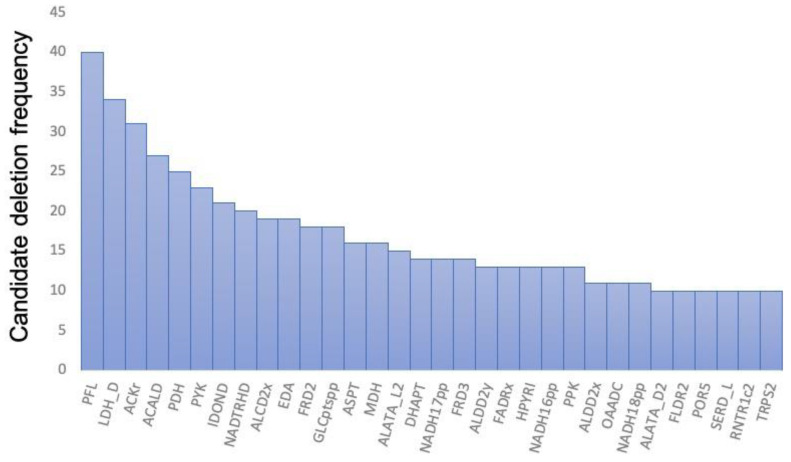
Top 32 reactions that were most frequently identified as candidates for deletion in the production of target compounds and their respective frequencies. A total of 32 reactions were selected in order of frequency of selection of 189 reactions that could have potentially been deleted for the production of 52 compounds. For example, the deletion of pyruvate-formate lyase (PFL), which was observed at the highest frequency, was required for 40 of the 52 compounds. The abbreviations, descriptions, and reactions are the same as in the iJO1366 model [24].

To evaluate the AERITH algorithm proposed herein, calculations were performed for each of the 81 compounds whose production could be confirmed using the iJO1366 model. These compounds were those for which FBA was performed using the compounds described as exchange fluxes in the model as the objective function, after which positive values were confirmed. The 81 compounds, their theoretical maximum production flux values, and the *OUR_min_* values are summarized in Supplementary Table S2. In this study, the upper bound of the exchange flux other than the targeted compound was set to zero. When AERITH was run without this condition, for example, 1,2-propanediol, hexanoate, or _L_-alanine were mainly produced, and sometimes no reaction-deficient combinations were proposed for the production of the target compounds. These compounds are not naturally produced by *E. coli*, i.e., they can only be produced by artificially enhancing genes related to their biosynthetic pathways [27,28,29,30,31]. Therefore, it is logical not to consider the conditions under which these compounds are produced naturally in the course of calculations to maximize growth. On the contrary, for the compounds known to be produced by *E. coli*, such as succinate, ethanol, formate, acetate, _D_-lactate [32,33,34], urea [35], hydrogen sulfide [36], and citrate [37], the upper bound of the exchange flux was set to 1000 (colored red in Table S2). The upper bound of the exchange flux of carbon dioxide was also set to 1000 for all calculations. Reaction deletions were not explored for 18 of the 81 compounds. Furthermore, for five of these eighteen compounds, a set of deletions that promoted target compound production could be identified when the *r* value was decreased from 0.95 to 0.90 (Supplementary Table S3). The more reaction deletions accumulated, the narrower the solution space of FBA became. By changing the parameter of *r*, one of the factors that determines the parameter *chg*, other varieties of combinations of reaction deletions for a target production can be found. There is no relationship between varying the *r* value and the set of reaction deletions obtained, but by setting the appropriate *r* value, a combination of fewer reaction deletions for high production of a target compound can be found. A set of proposed reaction deletions was summarized for each of the 68 compounds that spontaneously produced the target compound while maintaining growth maximization conditions (Supplementary Table S4). Effective combinations of reaction deletions for a metabolic model with more than 2000 reactions (such as iJO1366) were successfully obtained. The computation time required to identify the deletion combinations for a target compound was approximately 1 min, which was considerably shorter than that of the OptKnock method. Additionally, we were able to easily search for combinations of deletions in more than 20 reactions, which is more than the number of combinations that can be searched using OptKnock alone. The yield of each compound obtained by the combination of reaction deletions from AERITH execution was then determined by calculating the ratio of the maximum production flux of the target compound during AERITH execution to the flux of the target compound according to FBA, when the objective function was set to the target compound (maximum theoretical yield; Figure 2). The maximum number of compounds that could achieve more than 90% of the theoretical yield was 22. Furthermore, the number of compounds for which a set of deletions achieved more than 50% of the theoretical yield exceeded 80% of all target compounds (52/63 compounds), as demonstrated via piling curve analysis. The reactions of the required deletions were analyzed for the production of these 52 compounds. The number of reactions to be deleted for the production of these compounds was only 189, compared to 1688 for all candidate reactions (Supplementary Table S5). Additionally, the frequency of selection of 32 reactions of 189 reactions accounted for more than 50% of the total likelihood of being selected as deletion candidates. The top 32 types are illustrated in Figure 3. Deletion of the reactions involved in the synthesis of lactic acid (LDH_D, PYK, POR5), ethanol (PFL, PDH, ALCD2x, ALDD2x, ALDD2y), acetic acid (PFL, ACKr, ACALD, PDH), and succinic acid (FRD2, MDH, ASPT, FRD3) promoted the production of various compounds in *E. coli*. These compounds were byproducts produced specifically in oxygen-limited *E. coli* cultures. The reactions involved in the degradation and regeneration of ATP (PPK, RNTR1c2, ADK1) and those involved in redox reactions (NADTRHD, NADH17pp, NADH16pp, NADH18pp, and FLDR2) were also frequently selected as candidates for deletion, indicating that these reactions contribute to the supply of ATP and reducing power (NADH and NADPH), the latter of which acts as a cofactor for the production of target compounds. Although the OptKnock-based metabolic design method requires narrowing down the candidate reactions to be deleted [14], the inclusion of the 189 candidate reactions identified in this study may facilitate the identification of effective candidates for deletion when executing OptKnock.

The effectiveness of this algorithm was compared with that of other similar tools. Specifically, the results for acetate and succinate production using the iAF1260 model of *E. coli* were compared with those of GDBB (Table 1), a method developed by Egen and Lun [21], which can be used to rapidly search for many combinations of reaction defects in combination with OptKnock. In terms of acetate production, the method proposed in this study successfully identified highly productive reaction combinations by implementing three reaction deletions, and a higher yield could also be achieved with 17 reaction deletions than that of the GDBB method. The production yield of acetic acid was 88% (23.87/27.08). Furthermore, compared with the GDBB method, our proposed method successfully identified combinations of reaction deletions that were more productive under conditions of high growth rate. Therefore, the use of the proposed reaction-deficient strain in this method is expected to result in higher production per culture volume. Succinate production could not be confirmed with one or two reaction deletions using the proposed method in this study. In contrast, the GDBB method predicted instances with relatively high production yields based on one reaction deletion. This is because the GDBB approach uses bi-level optimization to maximize not only growth but also succinate production, while the method proposed in this study only maximizes growth. The byproducts ethanol and _D_-lactic acid were produced by deleting one and two reactions, while succinic acid production was observed only after deleting the three reactions involved in the synthesis of these two byproducts (Supplementary Table S6). Furthermore, the method proposed in this study is effective when the number of deletions is large, whereas OptKnock-based in silico screening is only effective for a small number of deletion combinations. For example, by removing five reactions, it was expected that both growth rate and production yield would be higher than those with GDBB. Additionally, the deletion of seven reactions enabled this method to achieve a succinic acid theoretical yield of 98% (14.14/14.49). Moreover, the results obtained with the method proposed herein were comparable with the results obtained by combining FastPros and OptKnock, as described by Ohno et al. [20]. A metabolic design with 10 reaction deletions was proposed for the optimization of _L_-Phe production. In this study, a metabolic design that favored _L_-Phe production was obtained by combining deletions in eight reactions (Table 2). The FastPros/OptKnock-based method achieved a _L_-Phe productivity of 58% of the theoretical yield, whereas our proposed method reached 86%. Additionally, the FastPros method required several hours to complete the necessary calculations, whereas our method successfully identified deletion combinations in less than a minute. To rule out the algorithm in this study being effective for the iJO1366 model of *E. coli* by chance, we further confirmed its effectiveness by implementing it for the following compound production models and comparing the results with those of Optknock [16,38,39]. The results for the *E. coli* model iJR904 with an additional 1,4-butanediol synthetic pathway are shown in Table S7, the results for the *Synechocystis* sp. PCC 6803 model iJN678 with a butanol biosynthesis pathway are shown in Table S8, and the results for 2,3-butanediol production using *Saccharomyces cerevisiae* model iMM904 are summarized in Table S9.

It is important to state both the reduction in computation time and the validity of the deletion sets obtained from the execution of the algorithms in this study for real production. In the production of succinic acid in *E. coli*, the algorithm used in this study predicts high production with five deletions in PFL, PDH, LDH_D, G6PDH2r, POX (11.647/14.489 of ideal yield) (see Table S6). This prediction is similar to the combination of six reaction deletions (*ldhA*, *adhE*, *ackA*, *pflB*, *mgsA*, and *poxB*) in succinate-producing strains shown in real experiments [40]. PFL corresponds to *pflB*, LDH_D to *ldhA*, and POX to *poxB*, respectively. PDH deletion stops the supply of pyruvate to acetyl-CoA, which is considered to be equivalent to a defect in acetic acid (*ackA*) and ethanol (*adhE*), which are synthesized from acetyl-CoA. Although the algorithm did not predict the possibility of lactic acid production bypassed by the *mgsA* reaction, it could be said that the algorithm was able to propose a combination of reaction deletions similar to an actual high-producing strain. A comparison of the deletion reactions performed for the construction of 2,3-butanediol-producing yeast [39] with the candidate deletions obtained with this algorithm showed interesting results. Ng et al. [39] constructed strains based on a combination of reaction deletions derived in Optknock and subsequently performed the corresponding reaction deletion because glycerol by-production was observed experimentally. This algorithm was able to predict the phenotype by accumulating the reaction deletions, and was able to propose the need for the reaction deletion (G3PD1ir, see Table S9).

Compared with the OptKnock-based method, the method described in this study showed a stepwise decrease in growth by selecting one deletion reaction. Moreover, target compound yields could also be improved. Therefore, researchers can select the appropriate combination of reaction deletions that satisfy the desired growth and productivity, after which bacterial strains can be developed for downstream experimental phases. This method tends to have a relatively large number of candidate deletion reactions because it is a stepwise method to increase the production of the target compound. When constructing the strain, the number of reaction deletions required may be reduced by evaluating relevant phenotypes via gene expression analysis [41,42], protein expression analysis [43,44], or flux analysis [45,46]. In recent years, an increasing number of studies have focused on designing, building, and testing microbial cells based on synthetic biology for the systematic and high-throughput production of desired compounds. Additionally, biofoundries have been established worldwide to make these efforts possible [47]. Given the importance of in silico design for the accurate development of useful microorganisms, the methods proposed in this study are expected to contribute greatly to the field of synthetic metabolic design.

## Figures and Tables

**Table 1:** Comparison between the results obtained using the method proposed in this study and the method described by Egen and Lun [21] for the production of acetate and succinate. The number of knockouts and the growth and production fluxes of the target compounds are described below. See Supplementary Table S6 for details of the deletion reactions.

Target	Number of Knockouts	This Study		Egen and Lun [21]	
		Biomass Flux (/h)	Target Flux (mmol/gDCW/h)	Biomass Flux (/h)	Target Flux (mmol/gDCW/h)
Acetate	1	0.34	11.14	0.12	12.10
	2	0.31	7.42	0.13	13.79
	3	0.26	16.14	0.05	15.12
	8	0.26	16.58	0.05	19.23
	17	0.11	23.87	-	-
Succinate	1	0.47	0.00	0.12	9.04
	2	0.36	0.00	0.10	9.26
	3	0.23	7.31	0.10	9.36
	4	0.22	6.68	0.06	9.60
	5	0.12	11.65	0.07	10.49
	6	0.12	11.65	0.09	10.61
	7	0.07	14.14	0.06	11.26
	8	0.07	14.22	0.07	11.53
	9	0.06	14.40	0.06	11.66
	10	0.06	14.40	0.06	11.74
	11	0.06	14.41	0.06	12.00
	12	0.05	14.43	0.06	11.91
	13	0.05	14.43	0.05	12.01
	14	0.05	14.43	0.05	12.01
	15	0.05	14.43	0.05	12.02
	16	0.05	14.43	0.05	12.04

**Table 2:** List of reaction deletions proposed by Ohno et al. [20] and those proposed in this study for the production of _L_-Phe. Both methods proposed three reaction deletion candidates in common. In addition to these three reactions, a minimum of four reactions required deletion in this study to confirm the production of _L_-Phe, with a production flux of 1.54 mmol/gDCW/h and a productivity of 36% of the maximum theoretical yield. Further deletion of transketolase (TKT1) was found to result in a production flux of 3.67 mmol/gDCW/h with an 86% yield.

This Study			Ohno et al. [18]		
Knockout Reaction		Target Flux (mmol/gDCW/h)	Knockout Reaction		Target Flux (mmol/gDCW/h)
Common	3 reactions				
3 reactions					
ALCD2x	Alcohol dehydrogenase				
PPC	Phosphoenolpyruvate carboxylase				
PYK	Pyruvate kinase				
Different	5 reactions			7 reactions	
PFL	Pyruvate-formate lyase		F6PA	Fructose 6-phosphate aldolase	
LDH_D	D-lactate dehydrogenase		G6PDH	Glucose 6-phosphate dehydrogenase	
ADK1	Adenylate kinase		PGCD	Phosphoglycerate dehydrogenase	
PPK	Polyphosphate kinase	1.54 (36 %)	GLYCD	Glycerol dehydrogenase	
TKT1	Transketolase	3.67 (86 %)	PTA	Phospho- transacetylase	
			NDH	NADH dehydrogenase	
			GLCNt	Gluconate transporter	2.46 (58 %)

## Data Availability

All data generated or analyzed during this study are included in this published article and its supplementary information files. AERITH is freely available at https://github.com/TomokazuShirai/AERITH_for_MATLAB.

## Supplementary Materials

The following supporting information can be downloaded at: https://www.mdpi.com/article/10.3390/biom12050620/s1, Table S1: Reactions not identified as deletion candidates; Table S2: Eighty-one compounds, their theoretical maximum production flux values, and their minimum oxygen uptake rate values; Table S3: A set of deletions that promoted target compound production were identified when the r value was decreased from 0.95 to 0.90; Table S4: A set of proposed reaction deletions for each of the 68 compounds that spontaneously produced the target compound while maintaining growth maximization conditions; Table S5: A list of the number of reactions to be deleted for the production of compounds; Table S6: Results of running AERITH for acetic acid and succinic acid production, respectively; Table S7: Comparison of the results of implementing AERITH and Optknock for the *E. coli* model iJR904 with an additional 1,4-butanediol synthetic pathway; Table S8: Comparison of the results of implementing AERITH and Optknock for the *Synechocystis* sp. PCC 6803 model iJN678 with a butanol biosynthesis pathway; Table S9: Comparison of the results of implementing AERITH and Optknock for 2,3-butanediol production using *Saccharomyces cerevisiae* model iMM904.
