# Supplementary material for: In Silico Design Strategies for the Production of Target Chemical Compounds Using Iterative Single-Level Linear Programming Problems
Source: Biomolecules. 2022 Apr 21;12(5):620. doi: 10.3390/biom12050620 (PMC9138359; doi:10.3390/biom12050620)
Supplement: Supplementary file 1 [file biomolecules-12-00620-s001.zip › Supplementary_Information.pdf]

Supplementary information

Supplementary Table S1

A total of 895 reactions from the iJO1366 model of *Escherichia coli* that were not candidates for deletion in this *in silico* screening are listed. The abbreviations, descriptions, and reactions are the same as in the iJO1366 model (Orth et al., 2011).

Supplementary Table S2

Theoretical maximum production flux (TrgtFlux) and oxygen consumption rate (o2consFlux, i.e., OUR_{min}) of 81 compounds whose extracellular production could be confirmed using the iJO1366 model. Each TrgtFlux value was calculated under the following conditions: objective function set to the target compound; lower limit of cell growth (μ_{min}) = 0.05/h. See Methods 2.2 for more details on the calculation. The abbreviations, descriptions, and reactions are the same as in the iJO1366 model (Orth et al., 2011).

Supplementary Table S3

List of compounds whose production could not be confirmed by performing AERITH. For the 18 compounds listed here, a valid set of deletion reactions at r = 0.95 could not be identified. However, for spermidine, L-cysteine, L-threonine, xanthosine, and 3-hydroxypropanoate, a set of reaction deletions that produced the respective compounds when calculated at r = 0.90 (s: success)

was explored. For the other compounds, varying r did not yield valid results (f: failure). The abbreviations, descriptions, and reactions are the same as in the iJO1366 model (Orth et al., 2011).

Supplementary Table S4

List of reaction deletions for each of the 68 compounds for which production was confirmed. Each sheet was named after the compound targeted for production. The growth rate and production flux of the target compound are listed in each sheet and the reaction deletions were multiplied from top to bottom. The abbreviations, descriptions, and reactions are the same as in the iJO1366 model (Orth et al., 2011).

Supplementary Table S5

List of frequencies of the deletion reactions required to produce the target compound. The frequency of reaction deletions required to achieve at least 50% of the theoretical yield of each of the 52 compounds is summarized. The reactions are listed from top to bottom in order of frequency. The abbreviations, descriptions, and reactions are the same as in the iJO1366 model (Orth et al., 2011).

Supplementary Table S6

List of deleted reactions when executing AERITH for acetate and succinate production using the iAF1260 model, and growth and production fluxes when the deletions are multiplied from top to bottom. The calculation conditions were established as in the iJO1366 model. See the

Methods for more details on our calculation procedures.

Supplementary Table S7

Comparison of the results of implementing AERITH and Optknock for the *E. coli* model iJR904 with an additional 1,4-butanediol synthetic pathway (Yim et al., 2011). The calculation conditions were established similar to those in the iJO1366 model. See the Methods for more details on our calculation procedures.

Supplementary Table S8

Comparison of the results of implementing AERITH and Optknock for the *Synechocystis* sp. PCC 6803 model iJN678 with a butanol biosynthesis pathway. The calculation conditions were same as described previously. See the Methods for more details on our calculation procedures.

Supplementary Table S9

Comparison of the results of implementing AERITH and Optknock for 2,3-butanediol production using *Saccharomyces cerevisiae* model iMM904. The calculation conditions were same as described previously (Ng et al., 2012). See the Methods for more details on our calculation procedures.

Reference

Orth, J.D.; Conrad, T.M.; Na, J.; Lerman, J.A.; Nam, H.; Feist, A.M.; Palsson, B.O. A comprehensive genome-scale recon-struction of Escherichia coli metabolism—2011. *Mol. Syst. Biol.* **2011**, *7*, 535.

Yim, H.; Haselbeck, R.; Niu, W.; Pujol-Baxley, C.; Burgard, A.; Boldt, J.; Khandurina, J.; Trawick, J.D.; Osterhout, R.E.; Stephen, R.; et al. Metabolic engineering of *Escherichia coli* for direct production of 1,4-butanediol. *Nat. Chem. Biol*. **2011**, *7*, 445–452.

Shabestary, K.; Hudson, E.P. Computational metabolic engineering strategies for growth-coupled biofuel production by *Synechocystis*. *Metab. Eng. Commun.* **2016**, *3*, 216–226.

Ng, C.Y.; Jung, M.Y.; Lee, J.; Oh, M.Y. Production of 2,3-butanediol in *Saccharomyces cerevisiae* by *in silico* aided metabolic engineering. *Micorb. Cell Fact.* **2012**, *11*, 68.
